# Targeting the IGF-1R: The Tale of the Tortoise and the Hare

**DOI:** 10.3389/fendo.2015.00064

**Published:** 2015-04-27

**Authors:** Caitrin Crudden, Ada Girnita, Leonard Girnita

**Affiliations:** ^1^Department of Oncology and Pathology, Cancer Centre Karolinska, Karolinska Institutet, Karolinska University Hospital, Stockholm, Sweden; ^2^Department of Dermatology, Karolinska University Hospital, Stockholm, Sweden

**Keywords:** IGF-1R, RTK, GPCR, biased signaling, cancer

## Abstract

The insulin-like growth factor type 1 receptor (IGF-1R) plays a key role in the development and maintenance of cancer. Since the first links between growth factor receptors and oncogenes were noted over three decades ago, targeting the IGF-1R has been of great interest. This review follows the progress from inception through intense pharmaceutical development, disappointing clinical trials and recent updates to the signaling paradigm. In light of major developments in signaling understanding and activation complexities, we examine reasons for failure of first line targeting approaches. Recent findings include the fact that the IGF-1R can signal in the absence of the ligand, in the absence of kinase activity, and utilizes components of the GPCR system. With recognition of the unappreciated complexities that this first wave of targeting approaches encountered, we advocate re-recognition of IGF-1R as a valid target for cancer treatment and look to future directions, where both research and pharmaceutical strengths can lend themselves to finally unearthing anti-IGF-1R potential.

## The Race Begins

### The tortoise starts slowly: Research interest develops

In 1983, two independent groups published their observations of sequence homology between an oncogene (Simian sarcoma virus oncogene, *v-sis*) and the platelet-derived growth factor (PDGF) (1, 2). Subsequently, numerous cellular oncogenes began to be described to be homologs of growth factors, growth factor receptors or of molecules within their signaling cascades: gp55, Bovine papilloma virus, SV40T antigen, among others. Orchestrating unrestricted cellular proliferation, it makes sense that oncogenes are found in the driving seat of cellular growth. Investigations and hypotheses that tyrosine kinase growth factor receptors (RTKs) were intimately involved in tumorigenesis and malignancy began to gain weight. The late eighties and early nineties seen research interest grow in this area, as multiple labs studied the PDGF-R and in particular the Insulin-like growth factor-1 receptor (IGF-1R) systems in *in vitro* models of human malignancies, starting with breast cancer (3, 4) and then extending to lung (5), prostate (6), bladder (7), and others (8–10).

### The hare shows interest: R-cells

The pivotal discovery in 1993 that mouse embryonic fibroblasts derived from embryos with a targeted disruption (homologous recombination) of the IGF-1R genes, named R-cells (11), were refractory to transformation, set of a tidal wave of excitement in the field of cancer therapeutics. Not only were these cells unable to be transformed by a panel of cellular oncogenes (SV40 T antigen (11), activated H-Ras (12), Raf, bovine papilloma virus (13) but importantly, the loss of this receptor had little effect on the cells normal *in vitro* growth (10% FBS). Mouse embryonic fibroblasts generated from wildtype littermates, as well as R-cells with the IGF-1R reinserted restored the transformation potential (14, 15). Animal models further propelled this wave, wherein mice and rat models given antisense IGF-1R strategies considerably decreased or abolished *in vivo* tumor growth yet had very little overall toxicity (16, 17).

### The hare’s sprint: Pharmaceutical development and clinical trials

**Figure 1:**
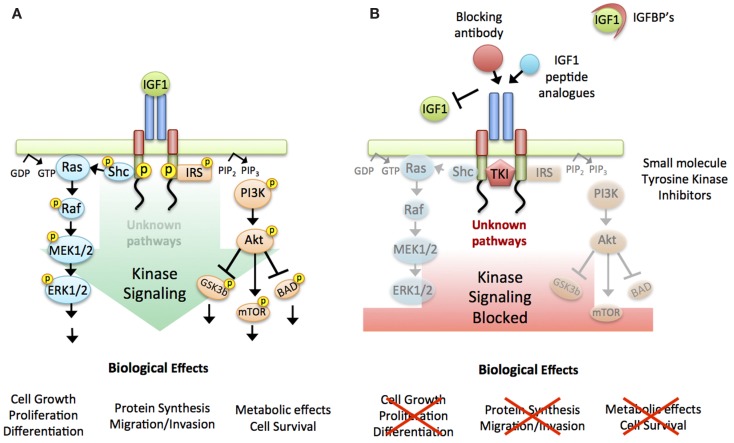
**Targeting the IGF-1R**. **(A)** Working model used to design agents targeting the IGF-1R: linear activation of all downstream signaling pathways triggered by ligand binding to the receptor and intrinsic kinase activation. Briefly: Ligand binding induces auto-phosphorylation of the receptor. This activated confirmation in turn activates two main downstream signaling cascades; mitogen-activated protein kinase (MAPK) and phosphoinositide 3-kinase (PI3K), ultimately leading to the biological effects of protein synthesis, cell survival, cell cycle progression, and proliferation. **(B)** IGF-1R targeting strategies: Two main approaches were taken to inhibit IGF-1R signaling, either by preventing the binding of the ligand to the receptor (IGFBPs, IGF1 peptide analogs or antibodies against the receptor or the ligand) or by blocking the receptor-kinase activation (small molecule tyrosine kinase inhibitors).

As antisense strategies do not work in humans, several approaches were undertaken in the late 1990s to target the IGF-1R in anti-cancer therapeutics, and with strong pre-clinical evidence multiple trials commenced. Over 30 drug candidates were developed and numerous clinical trials commenced (for current and regularly updated numbers see ClinicalTrials.gov) (18) as the IGF-1R became one of the most intensively investigated molecular targets in oncology. The therapeutics shared the common aim of inhibiting the kinase signaling cascade activated by the IGF-1R (Figure 1A), either by (i) prevention of ligand:receptor interaction e.g., through upregulation of the IGFBPs the natural IGF inhibitors (19), IGFs peptide analogs (20), or receptor/ligand blocking antibodies (21, 22), or (ii) IGF-1R signaling inhibition through e.g., small molecule tyrosine kinase inhibitors (23–26) (Figure 1B) Whilst most trials reported drugs to be well tolerated, actual clinical response was limited to a few cancer types (Ewing’s sarcoma, non-small cell lung cancer), not enough to maintain pharmaceutical interest. Overall, phase III trials were disappointing and agents were abruptly shelved [For in depth reviews of clinical trial results see (18, 23, 27–29)].

## The Hare Looses Interest and Takes a Nap: Why Did the Trials Fail?

**Figure 2:**
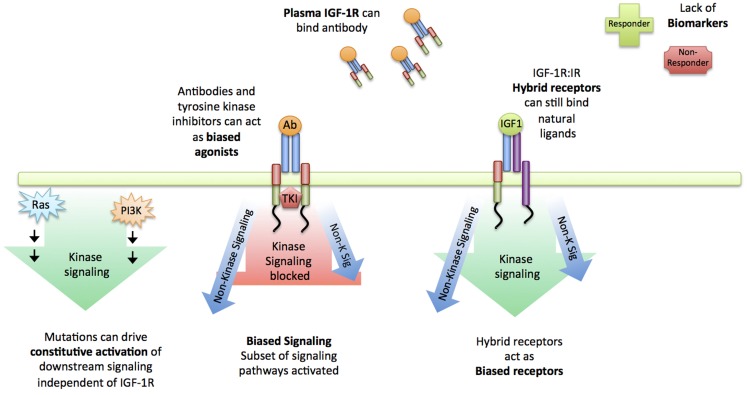
**Reasons for failure of IGF-1R targeting**. Summary of reasons suggested for the failure of IGF-1R targeting strategies: Constitutive activation of downstream signaling nodules such as Ras and PI3K drive signaling independent of the receptor. First-line targeted therapeutics such as blocking antibodies could be sequestered by plasma IGF-1R and in instances where they do reach the cell membrane, can act as biased agonists instead of antagonists, still activating a subset of signaling pathways. Similarly, IGF-1R:IR act as hybrid receptors, capable of binding natural ligands, activating signaling and likely lie under the radar of blocking IGF-1R antibodies. Altogether, a lack of biomarkers and insufficient understanding about the IGF-1R signaling complexities is likely to be the cause of clinical trial failure.

Many have postulated the reasons why anti-IGF-1R agents failed to live up to their hype (reviewed in (18, 23, 28, 29) (Figure 2). Whilst specific mutation of the IGF-1R is rarely reported in the literature, a large proportion of cancers carry a PI3K mutation (or PTEN deletion), constitutively activating Akt. In the instance of constitutive activation of a downstream signaling module such as Akt, the inhibition of the higher-level receptor will be futile, and given the rate of occurrence of this mutation across all cancer types, it is likely that this played a role (Figure 2). In much the same way, common mutations of the ERK pathway (Ras, Raf) will similarity constitutively activate the mitogen-activated protein kinase (MAPK) cascade, irrespective of IGF-1R inhibition (30) (Figure 2). In 2009, the importance of Insulin receptor substate-1 (IRS-1) was reported: in cells where IRS-1 is absent e.g., hematopoietic cells, IGF-1R stimulation leads to very little mitogenic signal activation or can actually induce differentiation, rendering IGF-1R targeting in these instances useless (31). Recently added to this list, is the investigation of plasma IGF-1R in cancer patients (32): anti-IGF-1R antibodies sequestered by circulating IGF-1R in the plasma could diminish any proposed therapeutic effect on cancer cells (Figure 2). Another important consideration is the close relationship between the IGF-1R and the Insulin receptor (IR). Many independent groups have demonstrated that the IR can replace mitogenic signaling in cells with low IGF-1R, and that the IGF-1R and IR can form hybrid receptors, capable of ligand binding, mitogenic signal activation and likely lie under the radar of IGF-1R designed antibodies (33–37) (Figure 2).

Along with cellular complexity outsmarting mono-therapy, it must be highlighted that the IGF-1R pharmaceutical race was different from others in its patient selection strategy. When viewed alongside the success stories in RTK therapeutics (e.g., HER2, C-kit therapeutics), the lack of any sort of patient selection, stratification or follow-up biomarker for therapeutic efficacy response in the case of IGF-1R, could very well have been a reason for failure. The flourish of excitement that a wonder drug lays within grasp hid the rational and well accepted need for careful patient selection. The IGF-1R trials included not only a wide range of cancer types, but also a broad range of molecular determinants and pre-trial treatment regimens (18) (Figure 2).

## The Tortoise Continues: Major Advancements in IGF-1R Understanding

### Signaling crosstalk

Whilst the canonical signaling schematics of the IGF-1R depict a ligand binding induced signaling cascade down through the MAPK and Akt pathways, it has long been recognized that intracellular signaling is much more network orientated than linear (Figure 1A). Indeed, at multiple levels throughout the canonical pathways, crosstalk to other receptors and other pathways can and indeed does occur, and one major outcome of the clinical trials was to reveal the hidden complexity of the IGF-1R signaling (Figures 1A,B) (28, 29). At the receptor level, the IGF-1R can not only form hybrid receptors with the IR (38), but the EGF-R has also been shown to have direct effects, with depletion affecting IGF-1R ubiquitination, degradation and signaling (39). In addition to other RTKs, there has been substantial work on the interaction with integrins, and their effect on IGF-1 signaling through RACK1 (40, 41) and SHPS1 and SJP2 (42, 43). Bidirectional crosstalk between the IGF-1R system and extracellular matrix components, such as SHP2 dephosphorylation of paxillin and FAK act as part of an integrin deactivation mechanism in cell migration (44). A system with such multilayered crosstalk and interaction offers plasticity and resilience to a “one-hit” targeting strategy.

### RNA pathway regulation

Alongside the explosion of interest in other aspects of cell biology, the non-coding-RNA regulation surrounding and controlling IGF-1R components is beginning to be pieced together. RNAs such as microRNAs and long-non-coding RNAs implement a further level of regulation around signaling pathways. This kind of regulation is illustrated in other pathways, such as p53 where a microRNA feedback circuitry has been identified and implicated in the pathogenesis of B-cell lymphocytic leukemia (45). Interestingly, RNA transcription resulting from one pathways activation as a method to regulate the signaling of a second pathway adds yet more feedback circuitry to the network, and whilst the relevance of this sort of RNA crosstalk has yet to be investigated for the IGF-1R, it hints at yet another level of signaling complexity.

### RTK:GPCR functional hybrid and the appreciation of biased signaling

A major recent advancement in IGF-1R biology is the challenge to the RTK functionality (28, 29). Whilst crosstalk between RTK and G-protein coupled receptor (GPCR) families have long been demonstrated and are well accepted (46), the functionality of the IGF-1R stood to be challenged by the demonstration that it suffices to fulfill all functional definitions of a GPCR. It was first reported that IGF-1R dependent MAPK signaling was sensitive to pertussis toxin, a toxin which uncouples the G-protein Gαi from its cognate receptor (47). The “mere crosstalk” argument to these experiments was thrown into dispute with the demonstration that in 3T3-L1 adipocytes, in basal state, Gαi and Gβ were associated with the IGF-1R and upon IGF-1 stimulation, Gβ was released and Gαi association increased (48). The second parallel came with the demonstration that, in much the same way as in GPCRs, β-arrestin was shown to be important in signal termination and receptor internalization at the IGF-1R (49, 50). To complete the story, in 2012 our lab demonstrated the only missing link to define IGF-1R as a functional RTK:GPCR hybrid: the same mechanism as in GPCRs for β-arrestin-receptor binding to GRK-dependent serine-phosphorylated sites (51). The functional GPCR signaling paradigm of ligand binding resulting in GRK phosphorylation, β-arrestin recruitment, signal termination and internalization, and the changeable signaling landscape afforded through biased agonism have all been demonstrated experimentally for the IGF-1R (29, 51–53). Altogether this strongly supports the updating of the IGF-1R from a prototypical RTK to an RTK:GPCR functional hybrid. Implications of such an updating highlight the evidence of non-tyrosine-kinase signaling and the resultant shortcomings of a tyrosine kinase inhibitor in this system (28, 29) (Figure 2).

In the field of GPCR biology, the paradigm of biased agonism is now fully accepted and describes the process by which a ligand:receptor pairing can selectively activate various downstream signaling pathways preferentially or to different degrees (54, 55). In a striking similarity, an established IGF-1R targeting antibody, in addition to its intended mechanism of action (kinase inhibition) acts as an IGF-1R/β-arrestin-biased agonist (52). Moreover, with identification of the human antimicrobial peptide LL-37 as an agonist for the IGF-1R, its ability to activate only the MAPK cascade and not the Akt cascade demonstrates this paradigm in action at the IGF-1R once again (53). The understanding of the functional selectivity of agonist/antagonist binding opens up many more therapeutic possibilities at the IGF-1R than the “OFF” or “ON” model, but also many more questions about the true complexity of the IGF system. It is now clear that the receptor can trigger signaling in the absence of the ligand (49, 51, 56), in the absence of kinase activity (56, 57) and be selective towards which pathway it activates (29, 56).

Unappreciated complexity, through the existence of plasma IGF-1R, hybrid receptors, pathway crosstalk, GPCR signaling components, RNA pathway regulation, lack of biomarkers, to name but a few, swamps the simple anti-IGF-1R targeted therapies in obvious failure (28, 29). However, the story does not end there. And whilst the “Hare” may have lost interest and had a nap, there has always been the “Tortoise”, and basic research on the IGF-1R has continued in labs across the world. Since the disappointing clinical trials and industry’s frustration and near abandonment, academia has slowly unearthed a plethora of novel understandings of how the IGF-1R signals (29, 36).

## The Future: Who Will Win the Race?

In the early days of growth factor and oncogene research, in 1988, a 22-year long study commenced following a particularly interesting group of patients, to investigate the role of IGF-1R in aging, diabetes and cancer development (58). A population of Ecuadorian individuals suffering from Laron syndrome, carried mutations in the growth hormone receptor (GHR) gene which leads to severe GHR and IGF-1 deficiency. As the dust settled on the IGF-1R trial disappointments, this study was published (2011), and the results lent yet more support to the fact that the underlying hypothesis held true. In Individuals (*n* = 22) with severe IGF-1 deficiency due to mutation in the GHR gene, cancer was not a cause of death in any of the subjects, yet it accounted for 20% of cancer deaths in non-affected relatives (58), furthermore they exhibited no cases of diabetes, compared to the Ecuadorian normal 5% level in control relatives. Moreover, a study looking at centenarians’ offspring, found that they had lower circulating IGF-1 bioactivity and a lower incidence of cancer (59). The IGF-1R and cancer link formed a few decades ago from sequence homology identification and *in vitro* validation is reinforced once again by epidemiological studies, which are now coming of age. Despite failure in our naive targeting attempts, it seems the underlying truth remains.

All postulations ultimately underline the fact that anti-IGF-1R therapeutic strategies were overly simplistic and insufficient to have any grand therapeutic effect given the complexity of the system. Whilst reductionist box-to-box schematics of signaling pathways are undeniably useful in the molecular and cell biology classroom, we must also to be aware that they are impossibly simplistic. Basic research requires simplistic beginnings, but if we intend to translate these findings into therapeutics, the limitations and realistic utility of these simple schemes must be appreciated, something that may not have been fully realized in the IGF-1R story.

The future of IGF-1R therapeutics may still lie ahead in much smarter designed, second/third generation targeting that recognizes and complements the true complexity of the system. To take advantage of those few success cases within the clinical trials, research effort into biomarkers will be of critical importance. Biased agonists, which specifically select a subset of signaling downstream of a given receptor have come into play largely in the field of GPCR therapeutics, the largest therapeutically targeted biological agents of all drugs in use clinically (60). The true extent of RNA regulation around the IGF-1R system is being built currently, and may yield many more alterations and targets. And importantly, lessons learnt from targeted therapeutics across the tumor biology arena, teach us the pitfalls of mono-targeting and so it is very likely IGF-1R targeted therapeutic success lies in a multiple targeted approach and overall system destabilization, or multi-modality treatment.

With the combination of academia’s unearthing of the true signaling complexities, and pharmaceutical industry’s drug development and trial expertise, we may just witness a re-awakening of shared interest and a re-writing of the tale, and just maybe the Tortoise and the Hare will finish the race together.

## Conflict of Interest Statement

The authors declare that the research was conducted in the absence of any commercial or financial relationships that could be construed as a potential conflict of interest.
